# Oral Squamous Cell Carcinoma Exosomes Upregulate PIK3/AKT, PTEN, and NOTCH Signaling Pathways in Normal Fibroblasts

**DOI:** 10.3390/cimb47070568

**Published:** 2025-07-19

**Authors:** Dijana Mitic, Milica Jaksic Karisik, Milos Lazarevic, Jelena Carkic, Emilia Zivkovic, Olivera Mitrovic Ajtic, Jelena Milasin

**Affiliations:** 1 School of Dental Medicine, University of Belgrade, Belgrade, Dr. Subotica 8, 11000 Belgrade, Serbia; dijana.trisic@stomf.bg.ac.rs (D.M.); milica.jaksic@stomf.bg.ac.rs (M.J.K.); milos.lazarevic@stomf.bg.ac.rs (M.L.); jelena.carkic@stomf.bg.ac.rs (J.C.); 2 Institute for Medical Research, National Institute of Republic of Serbia, University of Belgrade, 11129 Belgrade, Serbia; emilija.zivkovic@imi.bg.ac.rs (E.Z.); oliveram@imi.bg.ac.rs (O.M.A.)

**Keywords:** exosomes, microRNAs, oral cancer cells, dysplastic cells, magnetic sorting, TEM analysis, NTA, fibroblasts, gene expression

## Abstract

Exosomes, small extracellular vesicles secreted by various cell types, have gained significant attention in cancer investigations. Isolation and characterization of exosomes derived from DOK (dysplastic oral keratinocyte), SCC (squamous cell carcinoma) and HaCaT (normal skin keratinocyte) cell lines and microRNA profiling were conducted. Magnetic sorting was applied to obtain pure exosomes. Morphology and size were characterized by transmission electron microscopy and nanoparticle tracking analysis. Validation of membrane exosomal markers (CD9, CD63) was performed via Western blotting. MiR-21, miR-31, and miR-133 levels were analyzed in exosomes and parent cells by qPCR. Biological effects of the exosomes were tested by adding them to fibroblast cultures and determining the expression of relevant carcinogenesis markers by qPCR. Exosomes appeared as cup-shaped nano-sized particles, and there was no difference regarding particle diameter and concentration between the three types of exosomes. The oncogenic miR-21 was significantly upregulated both in SCC and SCC-derived exosomes compared to DOK and HaCaT cells and their respective exosomes. However, miR-31 unexpectedly showed the highest expression in normal cells and the lowest in HaCaT exosomes. MiR-133, the tumor suppressor miRNA, was downregulated in both SCC and DOK cells compared to normal (HaCaT) cells, while the opposite situation was observed in exosomes, with HaCaT cells showing the lowest levels of miR-133. The differences in exosome content were reflected in signaling pathway activation in exosome-treated fibroblasts, with SCC exosomes exerting the most potent effect on several cancer-related pathways, notably PIK3/AKT, PTEN, and NOTCH signaling cascades.

## 1. Introduction

Head and neck squamous cell carcinoma (HNSCC) is the sixth most prevalent cancer worldwide, with approximately 900,000 new cases and over 400,000 deaths each year [1]. Oral squamous cell carcinoma (OSCC) represents more than 90% of all oral cancers, with a higher incidence in males than females [2], and it shows a gradual increase in the younger population [3]. HNSCC develops due to a combination of various risk factors, such as tobacco and alcohol use, chronic inflammation, ultraviolet radiation (particularly for lip cancer), infections with human papillomavirus (HPV) or Candida, immunosuppression, genetic susceptibility, and diet [4,5]. Due to the complex multifactorial nature of this malignancy, investigation of underlying mechanisms driving the transition from normal tissue to cancer is vital for improving our understanding of OSCC initiation and progression.

Crosstalk between cells and their microenvironment occurs through direct cell-to-cell interaction or the release of soluble molecules, such as cytokines, chemokines, and growth factors [6,7,8]. However, in the last decade, a new paradigm has emerged, highlighting extracellular vesicle (EV) trafficking as a key mechanism of cell-to-cell communication [9,10]. EVs carry various cargos (proteins, RNA, DNA, and lipids), which can be transported between cells, facilitating intercellular communication at both paracrine and systemic levels [11]. EVs play multiple roles in various physiological processes, including normal development and tissue regeneration [12], but also in different pathologies. Recent studies have pointed to important connections between cancer and EVs, i.e., multiple aspects of cancer development and progression have been linked to EVs, making them promising candidates for use as biomarkers for cancer diagnostics and monitoring as well as therapeutic tools in anticancer treatments [11,13,14,15].

Extracellular vesicles (EVs) are membrane-bound structures secreted by active cells into the extracellular space. Based on their size, origin, and mechanism of formation, they are categorized into three main types: exosomes, microvesicles, and apoptotic bodies. Exosomes are the smallest, typically measuring 30 to 150 nm, derived from the endosomal system. They are formed when multivesicular bodies undergo inward budding and subsequently fuse with the plasma membrane to release the contents. Microvesicles, ranging from 100 to 1000 nm, are generated through direct outward budding of the plasma membrane. In contrast, apoptotic bodies, which are larger than 1000 nm, arise as a result of cellular fragmentation during apoptosis [16,17]. Among these, exosomes have become a prominent research subject in oncology. The mechanisms by which exosomes derived from tumor cells support tumor growth are being extensively studied. These exosomes act as information carriers, transmitting molecular and genetic signals from tumor cells to nearby or distant normal or aberrant cells. Tumor cells’ exosomes, often referred to as “oncosomes”, are capable of transferring oncogenes between tumor and normal cells [18]. These vesicles carry proteins, lipids, and nucleic acids in active form, influencing gene expression in recipient cells and shaping their behavior [19]. The cargo within exosomes is shielded from extracellular enzymes by its membrane, ensuring intact delivery to target cells. They are secreted into the extracellular environment after fusing with the plasma membrane [20]. Although the exact mechanisms governing cargo sorting and packaging of individual exosomes remain unclear, depending on parent cells [21], exosomes are increasingly being recognized as a key component of a sophisticated, organized intercellular communication network [22].

MicroRNAs (miRNAs) represent the most thoroughly studied group of short non-coding RNAs [23]. They control the expression of target genes at the post-transcriptional stage, mainly by binding to complementary regions in the 3′ untranslated region of target mRNAs [24]. Altered expression of numerous miRNAs has been shown in cancer, indicating either their oncogenic, tumor-suppressing, or even dual role, depending on the specific miRNA and cancer type [25]. Currently, exosomal miRNAs are attracting growing interest for their involvement in recruiting and reprogramming critical elements of the tumor microenvironment, but our understanding of their broader role is still incomplete [26,27]. Cancer-associated fibroblasts (CAFs) are integral components of the tumor microenvironment, significantly influencing tumor progression and metastasis. Emerging evidence highlights the pivotal role of exosomal miRNA secretion in the interplay between normal fibroblasts (NFs), CAFs, and cancer cells [28].

The aim of this study was to contribute to the understanding of the role of exosomes in OSCC pathogenesis by (1) comparing premalignant (dysplastic) and malignant cells’ exosomal miRNA expression profiles, (2) establishing a relationship with their respective parental cells’ miRNAs, and (3) testing their potential effects on normal fibroblasts. For this purpose, exosomes from oral squamous cell carcinoma (SCC), oral dysplastic (DOK), and adult human epidermal (HaCaT, which served as control) cell lines were isolated and characterized. The relative expression levels of two oncogenic (miR-21, miR-31), and one tumor suppressor (miR-133) microRNA were quantified in both the exosomes and their respective parent cells. Furthermore, the effects of these exosomes on signaling pathway activation were evaluated in normal fibroblasts to assess functional differences based on their cellular origin.

## 2. Materials and Methods

### 2.1. In Silico Analyses

#### MicroRNA Expression and GEO Dataset Analysis

Prior to in vitro studies, the expression of miR-21, miR-31, and miR-133a in oral squamous cell carcinoma has been examined using network analysis based on the GEO dataset obtained from the National Center for Biotechnology Information (NCBI) database (GSE168227). Data processing and visualization were performed utilizing Python 3.8. Specifically, miRNAs with a log2 fold change > 1 and an adjusted *p*-value < 0.05 were considered significantly expressed miRNAs. The −log10 of *p*-values was calculated for visualization on the *y*-axis, while log2 fold change (logFC) values were plotted on the *x*-axis.

### 2.2. Cell Cultures

Dysplastic oral keratinocytes (DOKs) (European Collection of Authenticated Cell Cultures, Salisbury, UK, 94122104) were cultured in DMEM/F12 supplemented with 10% fetal bovine serum (FBS) and 1% penicillin/streptomycin (all from Invitrogen, Thermo Fisher Scientific, Waltham, MA, USA). Additionally, cell culture medium was supplemented with 0.5 µg/mL hydrocortisone (Invitrogen, Thermo Fisher Scientific, Waltham, MA, USA). Cells were incubated at 5% CO_2_ at 37 °C.

The oral cancer cell lines SCC–25 (ATCC^®^, Manassas, VA, USA, CRL-1628™) and SCC-15 (ATCC^®^, CRL-1623 ™) were cultured in DMEM/F12, supplemented with 10% FBS, 100 U/mL of a penicillin–streptomycin solution, and 400 ng/mL of hydrocortisone. SCC-25 was cultured under standard conditions in a humidified atmosphere with 5% CO_2_ at 37 °C. The complete growth medium was changed every 2–3 days. Since there was no statistically significant difference in the behavior of the two SCC cell lines at any level (neither physico-chemical, nor in terms of mRNA or microRNA expression), at least in our experimental settings, we decided to merge the results for the two oral cancer cell lines.

Adult human epidermal cells HaCaT (ATCC^®^, CRL-2310) were cultured in DMEM/F12 supplemented with 10% FBS and 1% penicillin/streptomycin under standard conditions in a humidified atmosphere with 5% CO_2_ at 37 °C. The complete growth medium was changed every 2–3 days. These cells served as the control group in expression analyses.

In the final data presentation, three distinct cell groups were delineated: malignant cells (SCC-25 and SCC-15, merged), dysplastic keratinocytes (DOKs), and normal keratinocytes (HaCaT).

Normal fibroblasts (NFs) were obtained from patients who underwent surgical extraction of impacted third molars at the Clinic for Oral Surgery. All patients provided informed consent, and the study was approved by the Ethical Committee of the School of Dental Medicine, University of Belgrade (no. 36/19). Fibroblast isolation and cultivation were conducted as previously described [29]. NFs were treated with freshly isolated exosomes from SCC, DOK, and HaCaT cells and the respective conditioned medium. For this purpose, the medium was centrifuged for 6 min at 1700 RPM to remove cells and debris, and the supernatant was used to mimic co-cultures, i.e., to imitate a real tumor environment [30].

### 2.3. Exosome Isolation

When cells reached 90% confluency, they were washed with phosphate-buffered saline (PBS, Thermo Fisher Scientific, Waltham, MA, USA). After the washing process, DMEM/F12 supplemented with 1% penicillin/streptomycin was added to the flasks, and cells were incubated at 5% CO_2_ at 37 °C for 20 h in exosome-free medium (high-glucose DMEM (Thermo Fisher Scientific, Waltham, MA, USA) with 1% ABAM). The supernatant was collected from the flasks, and exosomes were isolated via magnetic-activated cell sorting (MACS) [31]. Exosome isolation with the MACS Exosome Isolation Kit CD63 (Miltenyi Biotec, Germany) was performed following the instructions of the manufacturer. Briefly, the supernatant was centrifuged at 300× *g* for 10′, 2000× *g* for 30′, and 10,000× *g* for 45′ consecutively to remove the cells, cell debris, and larger vesicles. The CD63-conjugated microbeads were added to supernatant (50 μL/2 mL) and vortexed, followed by incubation for 1 h at room temperature in the dark. The samples were separated inside the μ-column an d placed in the magnetic field of the μMACS separator. After separation, labeled vesicles were transferred to the tubes in isolation buffer. For Western blot analysis, vesicles were kept in exosome lysis and elution buffer. Samples were kept at −20 °C until further analysis.

### 2.4. Exosome Characterization

Transmission electron microscopy (TEM)*.* Exosome samples were diluted 1:100 in phosphate-buffered saline (PBS), applied to carbon-coated copper grids, and fixed with 2.5% glutaraldehyde for 10 min. The grids were then negatively stained with 1% phosphotungstic acid (pH ~7.0), air-dried, and examined using a Philips CM12 transmission electron microscope (Philips, Eindhoven, The Netherlands) equipped with an SIS MegaView III digital camera (Olympus Soft Imaging Solutions, Münster, Germany).

Protein quantification. Protein quantification with bicinchoninic acid protein (BCA) was conducted (Thermo Fisher Scientific, Waltham, MA, USA) [32]. Standards were prepared using serial dilutions of bovine serum albumin, from 0 to 200 µg/mL. Standard solutions and samples were put in a 96-well plate, and the working solution was added. Plates were incubated for 30′ at 37 °C. Absorbance was measured by using a microplate reader at 550 nm. The experiment was performed with 3 replicates.

Nanoparticle tracking analysis (NTA). Particle concentration and size distribution were assessed using the ZetaView^®^ QUATT system (Particle Metrix, Inning am Ammersee, Germany). Prior to each measurement series, the instrument was calibrated with polystyrene beads according to the manufacturer’s protocol. Samples were diluted in PBS (Thermo Fisher) up to 1:3000 to ensure optimal particle concentrations for NTA. Data acquisition and analysis were conducted using ZetaView software (version 8.05.16 SP3), with sensitivity set to 78% and shutter speed to 100. For each sample, measurements were recorded at 11 different positions. All experiments were performed in triplicate, and the results are reported as the mean values.

Purity ratio measurement. Sample purity was determined by calculating the ratio between particle count (obtained via NTA) and protein concentration (measured by BCA assay), as previously described [33,34,35]. The results are expressed as the number of particles (P) per microgram of protein (µg).

Western blot (WB). Protein extraction for WB analysis was performed following the instructions of the manufacturer. Briefly, magnetically labeled vesicles were flushed out from the columns with lysis and elution buffer and used for further Western blot analysis. The protein concentration was measured on a microvolume spectrophotometer (BioSpec–nano Microvolume UV–Vis Spectrophotometer; Shimadzu Scientific Instruments, Columbia, MD, USA). For WB, identical volumes of protein samples were run on polyacrylamide gels and transferred to the polyvinylidene difluoride membrane (GE Healthcare, Chicago, IL, USA). The membrane was blocked with 4% milk (Serva Electrophoresis GmbH, Heidelberg, Germany) for 1 h at room temperature (20 °C ± 2) and probed with primary antibodies to β-actin (Abcam, Cambridge, UK), CD9 (Elabscience, Wuhan, China), and CD63 (Elabscience, Wuhan, China, E-AB-53280). Peroxidase-conjugated goat anti-rabbit immunoglobulin (R&D Systems, Minneapolis, MN, USA) was used as a secondary antibody, and goat anti-mouse immunoglobulin (R&D Systems) was used for β-actin. The experiment was performed in triplicate. The protein levels were imaged with a ChemiDoc Imaging System (Bio-Rad Laboratories, Hercules, CA, USA), estimated by densitometric scanning of the blots using the Image Lab (Bio-Rad Laboratories, Inc. Version 6.0.0.25) software tool, and normalized to β-actin.

### 2.5. RNA Isolation and Reverse Transcription

Total RNA was extracted from the cells and exosomes using TRIzol Reagent (Invitrogen, Thermo Fisher Scientific), according to the manufacturer’s recommendations. The RNA concentration was measured on a microvolume spectrophotometer (BioSpec–nano Microvolume UV–Vis Spectrophotometer; Shimadzu Scientific Instruments, Columbia, MD, USA). An oligo d(T) primer and RevertAid First Strand cDNA Synthesis Kit (Thermo Fisher Scientific, Waltham, MA, USA) were used to synthesize cDNA from 2 µg of total RNA. For assessing the miR–21, miR-31, and miR-133 expression levels, RNA was isolated from DOK and SCC-25 cells and the corresponding exosomes. Total RNA was also isolated from normal fibroblasts according to the same procedure, followed by cDNA synthesis. The relative expression levels of *PIK3CA*, *AKT*, *PTEN*, *VEGFA*, *NOTCH1*, and *HES1* were determined by qPCR as previously described [33] (the list of primers is given in the Supplementary Materials Table S1). Furthermore, relative gene expression is presented as a relative fold change in comparison to the untreated fibroblast cells.

### 2.6. TaqMan microRNA Assay

TaqMan microRNA assay was performed to detect miR-21, miR-31, and miR-133 in both cells and exosomes isolated from cell cultures for expression comparison. Reverse transcription was conducted using the TaqMan™ MicroRNA Reverse Transcription Kit (The Applied Biosystems, Waltham, MA, USA) with 3 µL of 5× concentrated miRNA-specific primer. The thermal cycler settings included 16 °C for 30 min, 42 °C for 30 min, and 85 °C for 5 min, followed by cooling to 4 °C. Quantitative PCR (qPCR) was performed in 20 µL reactions with Universal PCR Master Mix and TaqMan 20× assays of miR-21 (ID 000397), miR-31 (ID 002279), and miR-133a (ID 002246) plus 2 µL of the reverse transcription product. The thermal cycler protocol consisted of 50 °C for 2 min and 95 °C for 10 min, followed by 40 cycles of 95 °C for 15 s and 60 °C for 60 s. RNU44 (ID 001094) was used as an internal control for miRNA. The experiment was performed in duplicate, in three independent experiments. Fold change was calculated using the formula Relative Quantity (RQ) = 2^−ΔΔCT^ based on threshold cycle (Ct) values.

### 2.7. Statistical Analysis

Data analysis was performed using the statistical software GraphPad Prism version 9.0 (GraphPad Software, Inc., La Jolla, CA, USA). The Kolmogorov–Smirnov test was used for assessing the normality of data distribution. To identify statistical differences between groups, a *t*-test and multiple *t*-test were applied, or a Mann–Whitney test, depending on the normality of distribution. The difference was considered statistically significant at *p* < 0.05.

## 3. Results

### 3.1. miRNA Network Analysis in OSCC

**Figure 1:**
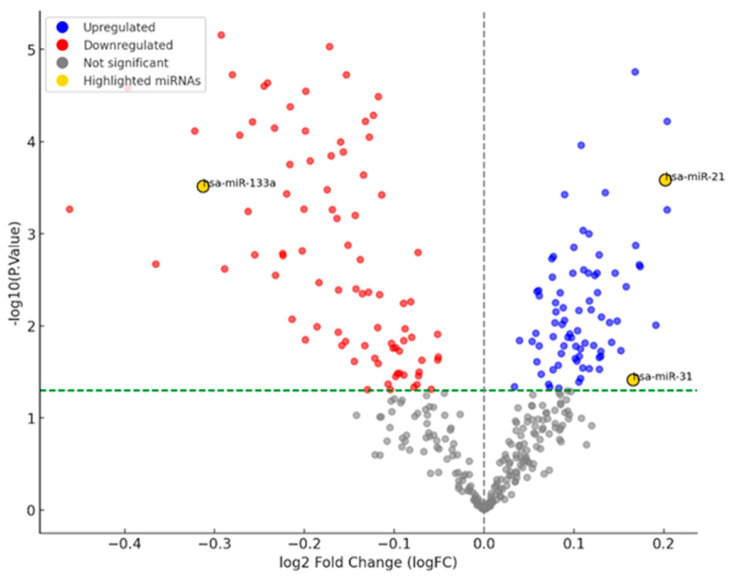
Volcano plot of differentially expressed miRNAs in OSCC.

The GSE168227 dataset contains a total of 369 miRNAs, which are visualized using a volcano plot to assess their differential expression in OSCC. Based on the criteria for significance (*p* < 0.05), the expression of 79 miRNAs was identified as significantly upregulated (blue points), and 86 miRNAs were identified as significantly downregulated (red points) in OSCC (Figure 1). In our analysis, miR-21 and miR-31 were found to be significantly upregulated, in contrast to miR-133a, which was significantly downregulated compared to controls.

### 3.2. Characterization of Exosomes Isolated from SCC, DOK, and HaCaT Cell Lines

**Figure 2:**
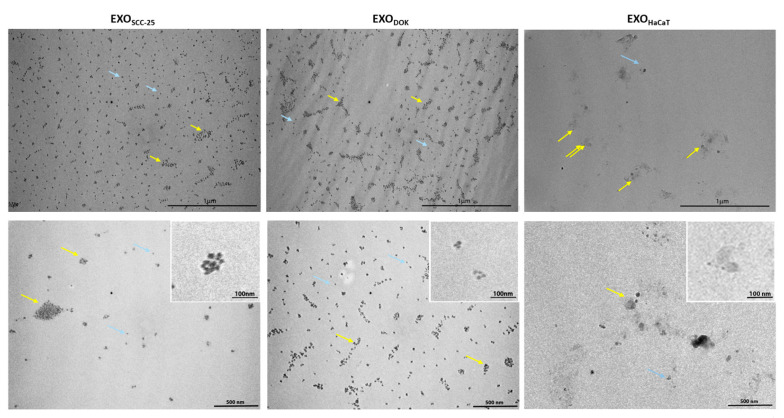
Representative transmission electron micrographs of exosomes from SCC (EXO_SCC_), DOK (EXO_DOK_), and HaCaT (EXO_HaCaT)_ cell lines. Negatively stained spherical structures are dispersed (blue arrows) or in clusters (yellow arrows), with some heavily decorated with magnetic beads.

Exosomes isolated using MACS appeared as spherical, nano-sized particles that were negatively stained. The size range of the isolated exosomes varied between 90 and 170 nm. There were no statistically significant differences between mean diameters of SCC exosomes (106.9 nm), DOK exosomes (107.3 nm), and HaCaT exosomes (121.3 nm). At the ultrastructural level, exosomes from all groups appeared as intact, cup-shaped particles or aggregated clusters, decorated with the beads. Additionally, the presence of magnetic beads was clearly distinguishable in the MACS-isolated samples (Figure 2).

**Figure 3:**
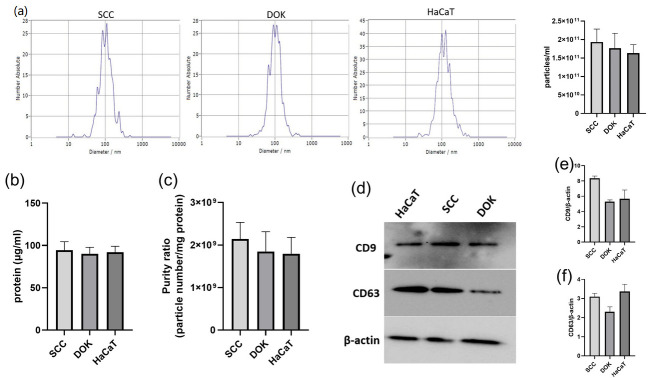
Characterization of isolated exosomes: (**a**) NTA analysis (particles/mL), (**b**) BCA protein quantification, (**c**) purity ratio of samples, (**d**) representative WB image of CD9 and CD63 expression in exosomes isolated from SCC, DOK, and HaCaT cell lines, (**e**) quantification of CD9 protein expression in isolated exosomes, and (**f**) quantification of CD63 protein expression in isolated exosomes; ns—not significant.

Similarly, particle concentrations did not show a statistically significant difference (1.93 × 10^11^ particles/mL vs. 1.80 × 10^11^ particles/mL vs. 1.60 × 10^11^ particles/mL in SCC, DOK, and HaCaT samples, respectively). The purity of samples was adequate, although SCC samples tended to be more homogenous. The exosomal surface marker CD63, as analyzed by Western blotting, exhibited slightly lower expression in exosomes isolated from DOK cells, whereas the CD9 marker showed slightly higher expression in SCC-derived exosomes. However, these differences were not statistically significant (Figure 3).

### 3.3. MicroRNA Expression in SCC, DOK, and HaCaT Cell Lines

**Figure 4:**
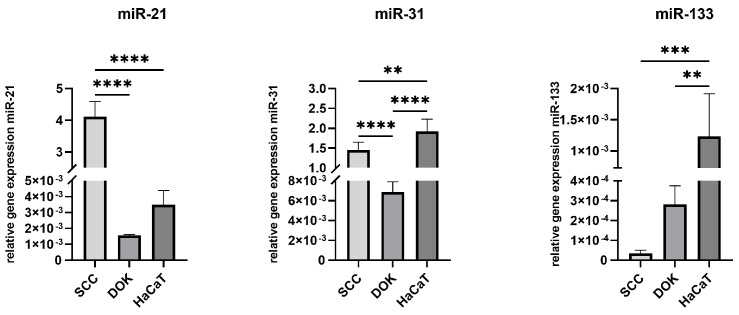
Relative microRNA expression analysis of miR-21, miR-31, and miR-133 in SCC, DOK, and HaCaT cell lines; ** *p* < 0.01, *** *p* < 0.001, and **** *p* < 0.0001.

MiR-21 and miR-31 were significantly upregulated in SCC cells in comparison to DOK cells, while the tumor suppressor miR-133 was significantly downregulated in malignant and dysplastic cells compared to normal HaCaT cells (Figure 4). Interestingly the other oncomir (miR-31) was overexpressed in HaCaT cells compared to both SCC and DOK cells.

### 3.4. MicroRNA Expression in SCC, DOK, and HaCaT Exosomes

**Figure 5:**
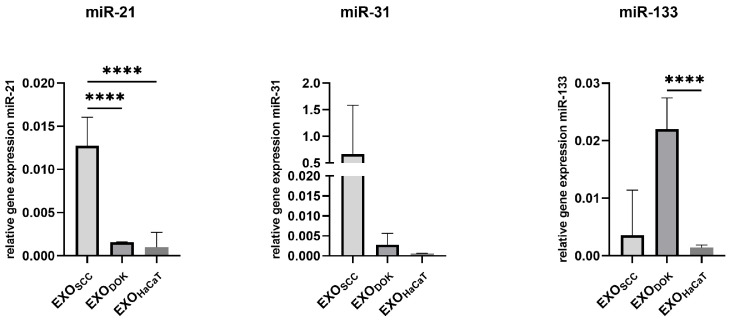
Relative microRNA expression analysis of miR-21, miR-31, and miR-133 in exosomes isolated from SCC, DOK, and HaCaT cell lines, **** *p* < 0.0001.

The expression pattern of miRNAs isolated from exosomes was somewhat different compared to the pattern found in the cells. MiR-21 was also upregulated in SCC exosomes compared to DOK and HaCaT exosomes, and that was also the case with miR-31, though without statistical significance. The levels of the tumor suppressor miR-133 were predictably low in SCC exosomes, but unexpectedly low in HaCaT cells and high in DOK cells (Figure 5).

### 3.5. SCC, DOK, and HaCaT Exosomes and Conditioned Medium Effects on Fibroblasts

**Figure 6:**
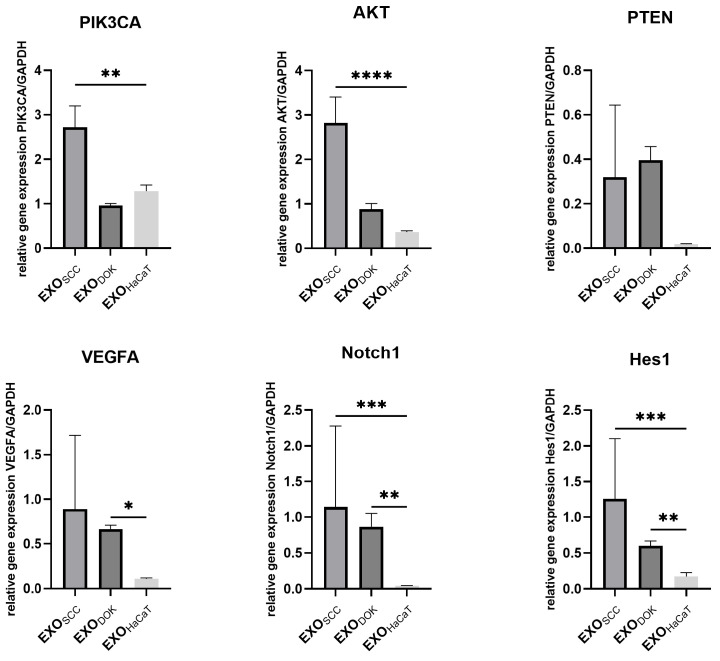
Expression analysis of crucial signaling pathways in fibroblasts treated with SCC exosomes (EXO_SCC_), DOK exosomes (EXO_DOK_), and HaCaT exosomes (EXO_HaCaT_); * *p* < 0.05, ** *p* < 0.01, *** *p* < 0.001, and **** *p* < 0.0001.

To further explore the effect of exosomes and conditioned medium (which, besides exosomes, contains cytokines, chemokines, growth factors, etc.), normal fibroblasts were subjected to 48 h cultures with (a) SCC exosomes/conditioned medium, (b) DOK exosomes/conditioned medium, and HaCaT exosomes/conditioned medium (Figure 6 andFigure 7). The relative expression levels of *PIK3CA*, *AKT*, *PTEN*, *VEGFA*, *NOTCH1*, and *HES1*, as the key cellular signaling pathways involved in cancer biology, angiogenesis, and cell survival/proliferation, were investigated to determine the effects of SCC, DOK, and HaCaT products on fibroblasts.

Generally, EXO_SCC_ displayed the highest impact on the signaling pathways that were examined. *PIK3CA*, *AKT*, *NOTCH,* and *HES1* were significantly upregulated in fibroblasts treated with EXO_SCC_ compared to fibroblasts treated with EXO_HaCaT_. Similarly, CM_SCC_ also induced considerable overexpression of *AKT*, *VEGFA,* and *HES1* in treated fibroblasts compared to the control.

EXO_DOK_ exerted a comparatively more modest effect on the analyzed signaling pathways. Specifically, DOK-derived exosomes induced moderate upregulation of *VEGFA*, *NOTCH*, and *HES1*, in contrast to the stronger activation observed with EXO_SCC_. In parallel, CM_DOK_ treatment led to increased expression of *PTEN* and *VEGFA*, along with downregulation of *PIK3CA.*

**Figure 7:**
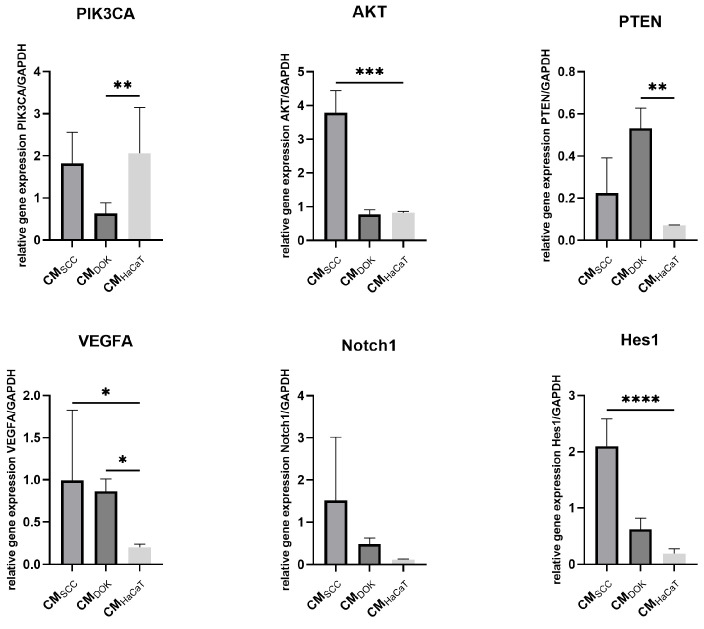
Expression analysis of crucial signaling pathways in fibroblasts treated with SCC conditioned medium (CM_SCC_), DOK conditioned medium (CM_DOK_), and HaCaT condition medium (CM_HaCaT_); * *p* < 0.05, ** *p* < 0.01, *** *p* < 0.001, and **** *p* < 0.0001.

## 4. Discussion

At present, miRNAs, especially exosomal ones, are gaining significant interest due to their role in recruiting and reprogramming key elements of the tumor microenvironment [26]. It has been previously shown that certain miRNAs are retained within cells while others are secreted in exosomes, indicating selectiveness of the process by which miRNAs are packaged into small vesicles [34,35]. Exosomal miRNAs that are selectively transported from cancer cells to the surrounding environment could play an important role in the malignant behavior of tumors. This prompted us to try to shed some new light on the relationship between microRNAs that are secreted through extracellular vesicles and microRNAs contained in the parent cells and to explore the differences existing in this respect between premalignant (dysplastic) and malignant cells. For this purpose, following their isolation, we first performed characterization of the extracellular vesicles from the DOK and SCC cell lines’ cultures. No noteworthy difference between DOK and SCC exosomes could be observed in terms of their size and concentration (nor was there any difference compared to HaCaT exosomes). We then performed qPCR analysis of specific miRNAs to compare the expression levels between oral carcinoma and dysplastic cells, as well as between the exosomes secreted by these cell lines.

One of the most widely recognized miRNAs in malignancies is miR-21, an oncomiR linked to several cancers, including HNSCC, esophageal squamous cell carcinoma, gastric carcinoma, lung cancer, colorectal cancer, breast cancer, and pancreatic cancer [36,37,38,39,40]. Increased levels of miR-21 are strongly associated with poor prognosis in various malignancies, owing to its oncogenic functions in promoting cell proliferation, migration, and invasion [33,37]. Our study first showed a significant increase in miR-21 levels in malignant (SCC) compared to premalignant/dysplastic (DOK) cells and normal (HaCaT) cells. This finding is fully in agreement with the microRNA expression GEO dataset analysis in OSCC. MiR-21 was also notably upregulated in exosomes derived from cancer compared to exosomes from dysplastic and normal cells. The consistent upregulation of miR-21 in SCC highlights its potential as a therapeutic target, where sponging strategies could help correct aberrant gene expression profiles in oral cancer. Previous studies have observed increased levels of miR-21 in oral premalignant lesions in comparison to normal oral mucosa, suggesting that changes in miR-21 may occur relatively early on in OSCC development [41,42].

A study using next-generation sequencing analyzed small RNA from parental cells and exosomes derived from laryngeal squamous cell carcinoma and showed that approximately 70% of all small RNAs belonged to miRNAs in both the parental cells and their corresponding exosomes. However, only about 5% of miRNAs were detected in both cells and their exosomes, while over 50% of miRNAs were only detected in exosomes, further supporting the idea that the packaging of miRNAs into exosomes is a selective process [43]. These selectively packaged exosomal miRNAs could enhance the malignant characteristics of tumors and be the crucial players in cell-to-cell communication between tumor cells and tumor and stromal cells. The most prevalent miRNA in cells was miR-21-5p, while in exosomes it ranked in the third place [43]. Our results support this observation of overall upregulation of miR-21, with high expression levels found both in oral carcinoma cells and in their respective exosomes. In our study, relative expression of miR-31, also classified as an oncomiR, was significantly upregulated in cancer compared to dysplasia cells. SCC exosomes tended to show higher expression of miR-31 than DOK and HaCaT exosomes but without reaching statistical significance. Unexpectedly, HaCaT cells exhibited the highest levels of miR31, which may be attributed to the high intrinsic capacity of keratinocytes to proliferate, differentiate, and migrate [44]. MiR-31 is known to be one of the most commonly altered microRNAs, along with miR-21, in several human cancers, including OSCC, and previous research has pointed to its role in tumorigenesis and OSCC development [45,46,47,48]. Typically, miR-31 is upregulated during malignant transformation of cells [49] and, among other factors, has been linked to epithelial-to-mesenchymal transition (EMT), which plays a crucial role in cancer development, metastasis, and progression [50,51]. Several studies have suggested that EMT could be a key mechanism by which miR-31 influences OSCC [52,53,54]. Upregulation of miR-31 was found in oral premalignant epithelium compared to normal mucosa [53], and a recent study showed that exosomes secreted by mesenchymal stem cells from oral leukoplakia with dysplasia stimulated proliferation, migration, and invasion similarly to exosomes derived from oral carcinoma cells [55]. It has been suggested that exosomes secreted by residual cells from oral leukoplakia could contribute to recurrence and potentially accelerate the malignant transformation process.

Contrary to the previously mentioned two microRNAs that possess an oncogenic potential, miR-133 in OSCC mostly behaves as a tumor suppressor [56]. It is involved in a range of biological processes, such as proliferation, apoptosis, autophagy, etc. Downregulation of miR-133 in malignant cancers is often associated with poor prognosis [57,58,59,60]. However, only a few studies have focused on the miR-133-dependent inhibition of cancer progression in oral and esophageal squamous cell carcinoma [61,62]. Research on esophageal and oral cancers has suggested that upregulated miR-133 inhibits EMT [63]. Our previous study has shown downregulation of miR-133 in oral cancer stem cells (CSCs), and following induced differentiation of CSCs, there was restoration of normal levels of miR-133 [56]. Another study indicated that stable expression of miR-133 in oral cancer cells was associated with an enhanced EMT phenotype, which correlated with increased migratory potential [64]. The results of the present study point to the noteworthy downregulation of miR-133 in cancer and dysplastic cells, compared to normal cells, consistent with its tumor suppressor role. Surprisingly, it was upregulated in DOK-derived exosomes when compared to normal (HaCaT) cells (alternatively, we could say that the levels of miR-133 in HaCaT exosomes were unexpectedly low). In the study of Yang et al., exosomes isolated from lung cancer cell lines were transfected with miR-133a-3p to explore its impact. The overexpression of miR-133a-3p delivered via exosomes enhanced the proliferation, angiogenesis, migration, and invasion of cancer cells both in vitro and in vivo [65]. These results are somewhat in line with the elevated levels of exosomal miR-133 found in DOK-derived exosomes, despite conflicting with the tumor suppressor status of this miRNA. Additional research is definitely required to better understand the role of miR-133 in the progression of oral carcinoma, as well as the potential contribution of exosome-delivered miR-133 in this process.

Functional assays demonstrated that exosomes derived from SCC cells exerted a significant influence on normal fibroblasts by activating key tumorigenic signaling pathways. Specifically, the PI3K/AKT pathway was markedly upregulated following EXO_SCC_ treatment. This activation is consistent with the elevated levels of miR-21 and miR-31 detected in both SCC cells and their secreted exosomes, i.e., in line with studies that have demonstrated that miR-21 and miR-31 upregulation contributes to AKT pathway activation in oral cancer [66,67]. We also observed miR-21 functional interplay with the Notch signaling pathway, as fibroblasts treated with EXO_SCC_ exhibited increased expression of both *NOTCH1* and its canonical target *HES1*. The effects of EXO_DOK_ on fibroblasts were somewhat milder compared to EXO_SCC_ in the sense that they did not activate the PI3K cascade. Expression levels of these markers (*PIK3A* and *AKT*) were similar to normal cells’ levels. This might be due to lower levels of miR-21 and miR-31 in DOK exosomes than in SCC exosomes. Yet DOK_EXO_ influenced Notch and VEGF pathways, i.e., upregulated them. Surprisingly, both SCC and DOK exosomes caused upregulation of *PTEN* in treated fibroblasts compared to the effect of normal cells (HaCaT) on fibroblasts, although without statistical significance. This marked downregulation of *PTEN* in the presence of HaCaT-derived products may be attributed to the known influence of epithelial cells on fibroblasts, which includes reduced *PTEN* expression that further leads to altered fibroblast behavior, such as increased proliferation and enhanced collagen production [68].

It must be underscored that the conditioned medium of both SCC and DOK showed only partial overlapping with the effects of the respective exosomes on normal fibroblasts, but at least no contradictory results were obtained. The only surprising finding was the upregulation of PIK3CA in fibroblasts treated with CM_HaCaT_, and this might be due to low levels of HaCaT miR133, which is a PIK3CA inhibitor [69].

A more comprehensive study covering additional cell lines and primary cultures, as well as other microRNAs, could offer deeper insights into the impact of exosomes’ cargo on cancer biology and potential therapy. In addition, the expression of different genes included in the present study should also be analyzed at the protein level.

## 5. Conclusions

Our study confirmed differences in the cellular and exosomal microRNA profiles of oral premalignant and malignant cell lines. Exosomes derived from both SCC and DOK cells modulated key tumorigenic signaling pathways in recipient normal cells, with the effects being more pronounced following treatment with SCC-derived exosomes.

## Data Availability

The original contributions presented in this study are included in the article/Supplementary Materials. Further inquiries can be directed to the corresponding author.

## Supplementary Materials

The following supporting information can be downloaded at https://www.mdpi.com/article/10.3390/cimb47070568/s1.

## Abbreviations

DOK	dysplastic oral keratinocyte
SCC	squamous cell carcinoma
NF	normal fibroblast
CAF	cancer-associated fibroblast
CM	conditioned medium
GEO	gene expression omnibus
EMT	epithelial-to-mesenchymal transition
CSC	cancer stem cell
OSCC	oral squamous cell carcinoma
