# Supplementary material for: Oral Squamous Cell Carcinoma Exosomes Upregulate PIK3/AKT, PTEN, and NOTCH Signaling Pathways in Normal Fibroblasts
Source: Curr Issues Mol Biol. 2025 Jul 19;47(7):568. doi: 10.3390/cimb47070568 (PMC12293655; doi:10.3390/cimb47070568)
Supplement: Supplementary file 1 [file cimb-47-00568-s001.zip › cimb-3516320-supplementary.pdf]

Table S1. Primer Sequences used in the study.

GENE	DIRECTION	SEQUENCE
**AKT**	Rv	5'GCAGAGAGGTAATCAGCACCAA 3'
	Fw	5'GCAAAGCAGGAGTATAAGAAAGGAA3'
**PIK3CA**	Rv	5'AAGTGGATGCCCCACAGTTC 3'
	Fw	5' TTACCCTCTTCTGCCGGAGG 3'
**Hes1**	Rv	5'CCGCGAGCTATCTTTCTTCA 3'
	Fw	5' TCAACACGACACCGGATAAA 3'
**Notch1**	Rv	5'CCACGAAGAACAGAAGCACA 3'
	Fw	5'AGCCTCAACATCCCCTACAA 3'
**PTEN**	Rv	5'GGGAATAGTTACTCCCTTTTTGTC 3'
	Fw	5'ACCCACCACAGCTAGAACTT 3'
**VEGFA**	Rv	5' GGCAACTCAGAAGCAGGTGA 3'
	Fw	5' GGGAGCTTCAGGACATTGCT 3'
**GAPDH**	Rv	5' CCC TGT TGC TGT AGC CAA ATT CGT 3'
	Fw	5' TCA TGA CCA CAG TCC ATG CCA TCA 3'
